# Supplementary figures and images for: Tissue and cellular tropism of elephant endotheliotropic herpesvirus (EEHV)1A in hemorrhagic disease
Source: PLoS One. 2025 Sep 2;20(9):e0330631. doi: 10.1371/journal.pone.0330631 (PMC12404477; doi:10.1371/journal.pone.0330631)

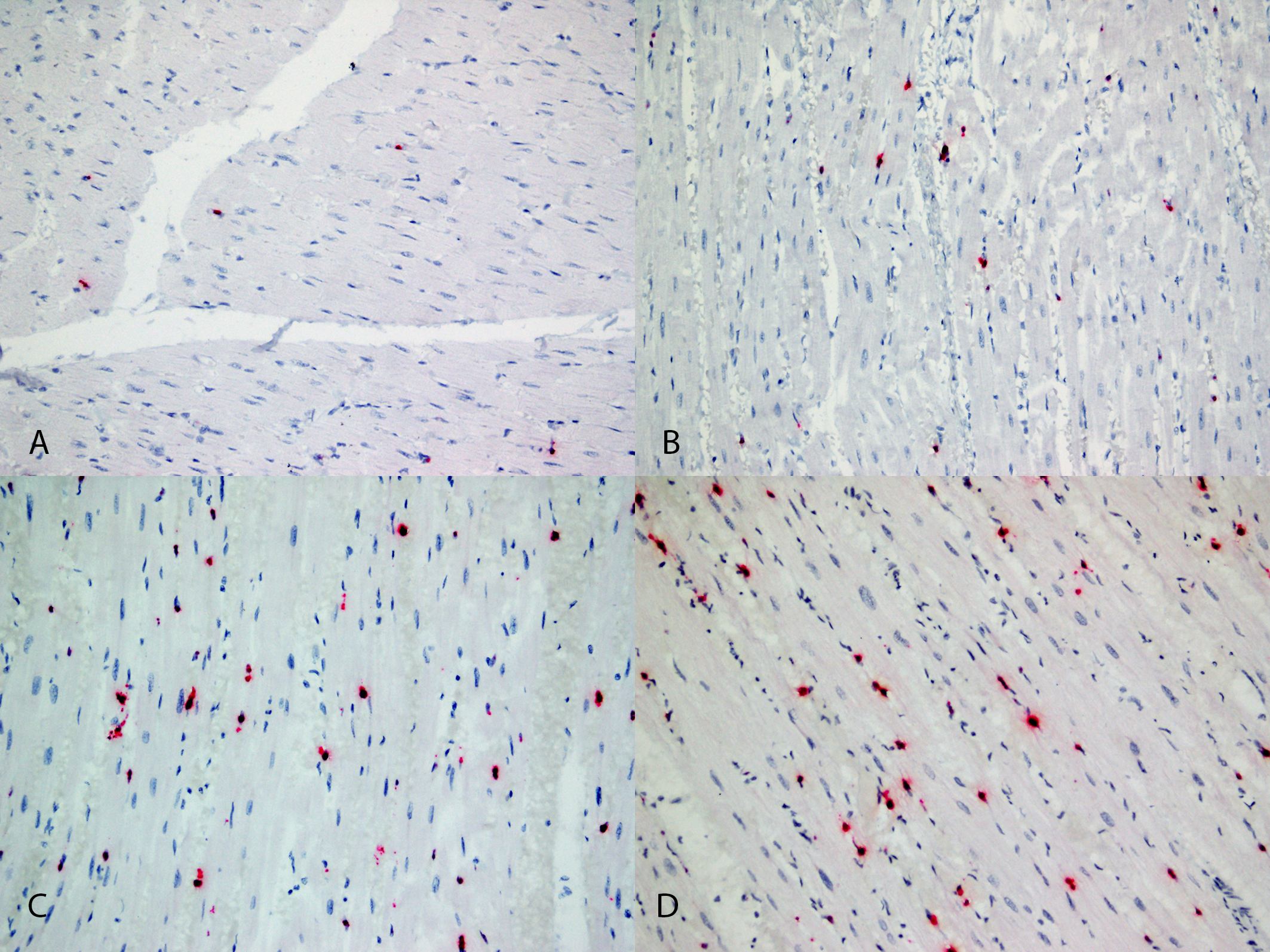

Supplement: S1 Fig — A. Myocardium from Case 4 hybridized with the EEHV1A terminase probe. Less than 10 endothelial cell nuclei in the 200x magnification field have positive in situ hybridization (ISH) signal represented by red staining. Findings are consistent with grade 1 signal. B. Myocardium from Case 5 hybridized with the EEHV1A terminase probe. Greater than 10 but less than 21 endothelial cell nuclei in the 200x magnification field have positive ISH signal. Findings are consistent with grade 2 signal. C. Myocardium from Case 1 hybridized with the EEHV1A terminase probe. Greater than 20 but less than 30 endothelial cell nuclei in the 200x magnification field have positive ISH signal. Findings are consistent with grade 3 signal. D. Myocardium from Case 2 hybridized with the EEHV1A terminase probe. Greater than 30 endothelial cell nuclei in the 200x magnification field have positive ISH signal. Findings are consistent with grade 4 signal. (TIF) [file pone.0330631.s001.tif]

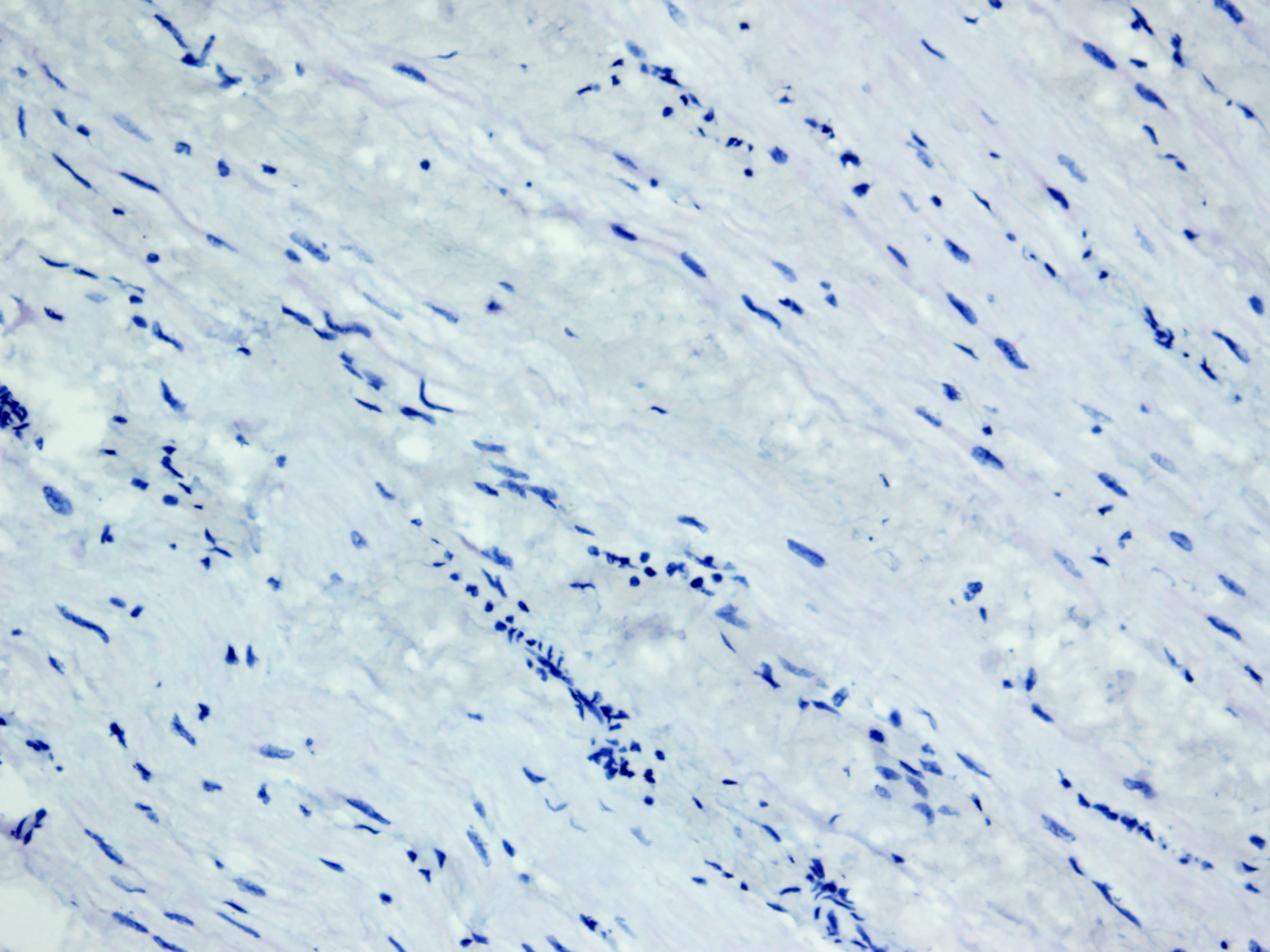

Supplement: S2 Fig — No positive (red) in situ hybridization signal is visible in cardiomyocytes, vascular smooth myocytes, endothelial cells or any other cells represented in the image. (TIF) [file pone.0330631.s002.tif]

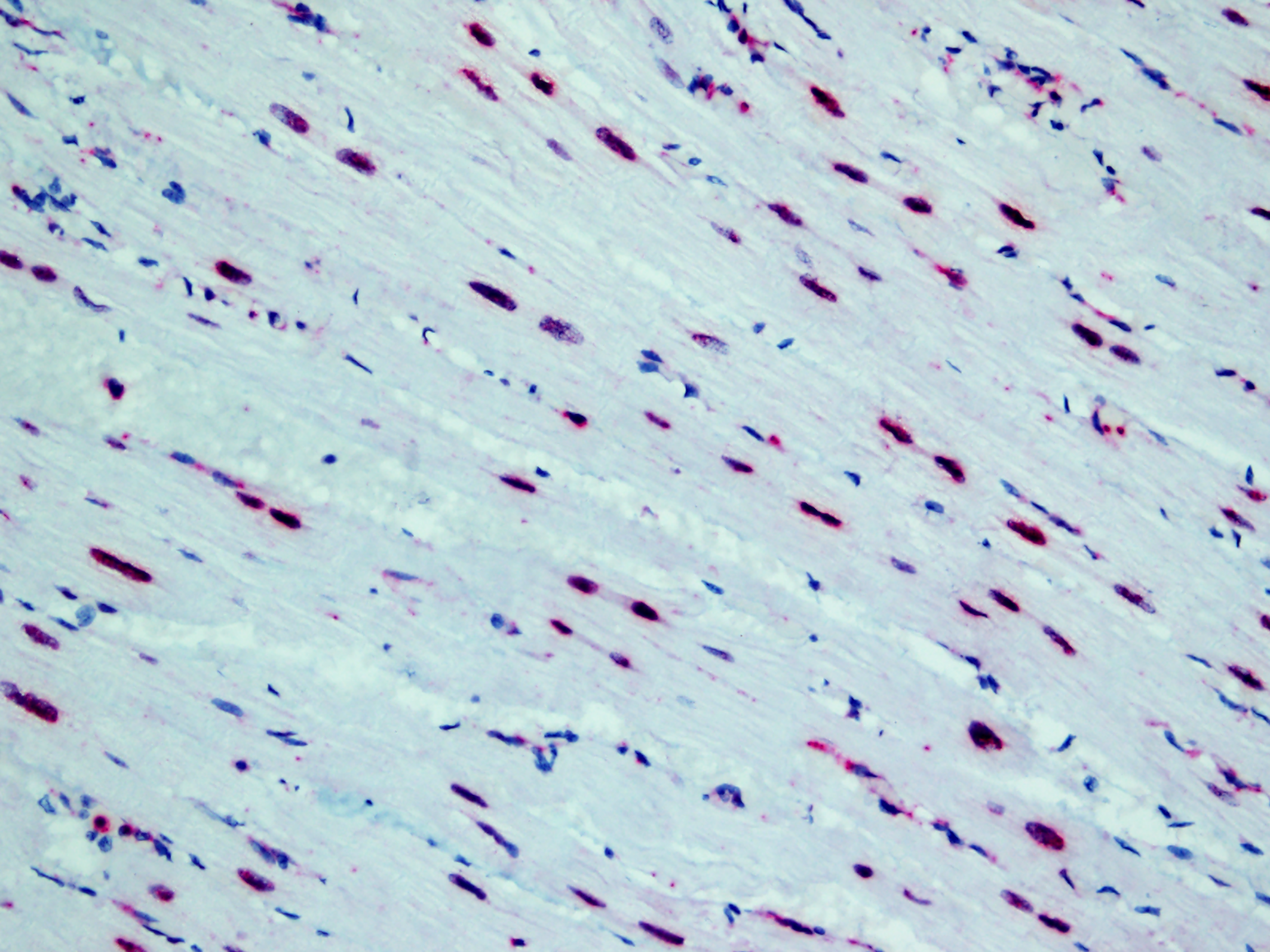

Supplement: S3 Fig — Positive (red) in situ hybridization signal is visible in multiple locations including nuclei of cardiomyocytes and cytoplasm of cardiomyocytes, endothelial cells, vascular smooth myocytes and some circulating leukocytes. (TIF) [file pone.0330631.s003.tif]
